# CAR-T cell therapy: potential for paediatric brain tumours—an update

**DOI:** 10.1007/s11060-026-05604-5

**Published:** 2026-08-28

**Authors:** Alexandra Zehner, Benjamin Draper, Darren Hargrave, Laura K. Donovan, John Anderson

**Affiliations:** UCL Great Ormond Street Institute of Child Health, University College London, London, UK

**Keywords:** Chimeric Antigen Receptor, CAR-T, Paediatric, CNS-tumour

## Abstract

Central nervous system (CNS) tumours are the deadliest cancer for children and currently present limited treatment options. Chimeric antigen receptor (CAR)-T cell therapies have emerged as an innovative approach supported by encouraging clinical results. Current clinical trials using CAR-T cells in the treatment of paediatric CNS cancers differ in a number of variables, including the CAR-T cell route of delivery, presence of lymphodepletion, identified target antigen, and CAR engineering features. Considering early learnings across these areas is an essential step to developing more effective treatment options, especially given the challenges of immunosuppressive tumour microenvironments, various toxicities, CAR-T cell exhaustion, and tumour antigen heterogeneity. In sum, while there is a need for continued innovation, CAR-T cells represent a promising treatment approach for this devastating category of diseases.

## Introduction

Central nervous system (CNS) tumours are the most prevalent solid cancer in the paediatric population [1], and within the past decade, have surpassed leukemia as the deadliest cancer for children 19 and younger [2]. This backdrop unwaveringly points to the necessity of innovative treatment options.

**Fig. 1:**
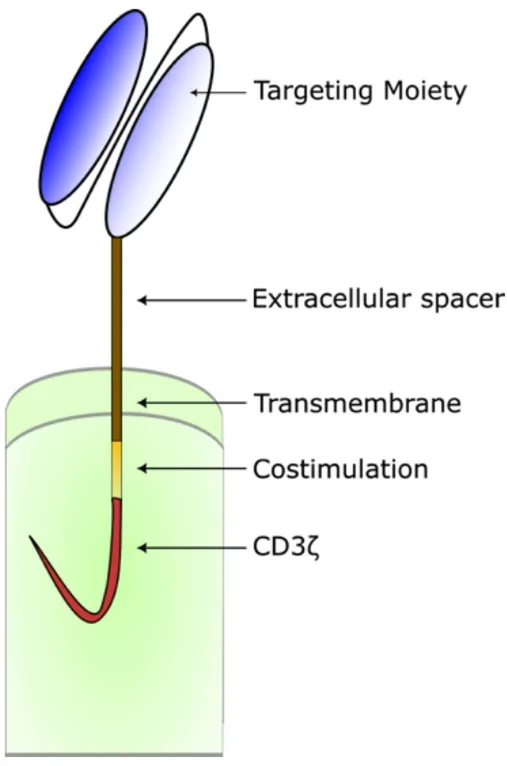
Second generation CAR. Second-generation CARs consist of four domains: a targeting moiety, often an ScFv derived from an antibody; a spacer/transmembrane that projects the targeting moiety away from the T-cell and can confer oligomerisation; a costimulation domain, commonly derived from 4-1BB or CD28 intracellular domains; an ITAM containing domain to confer T-cell activation, most commonly derived from CD3ζ [7]

Currently, limited therapy options exist for paediatric CNS tumours, with a dismal prognosis for many histologies. Standard approaches consist of surgery, radiation therapy, and chemotherapy. The intensity of these therapies, particularly when applied to a paediatric population, confers lifelong risk of severe disabilities. Immunotherapy in the form of checkpoint inhibitors [3, 4] and vaccine studies [5] has emerged as an exciting therapeutic opportunity but shown limited efficacy in this disease setting. Monoclonal antibodies have also shown limited benefit; for example, I-8H9 radioimmunotherapy targeting B7-H3 has been extensively investigated with no clear evidence of efficacy [6]. However, as targeted approaches offer exciting potential to reach precarious structures while limiting off-target toxicity, additional immunotherapy avenues are worth pursuing, including chimeric antigen receptor (CAR)-T cell therapies (Fig. 1).

**Fig. 2:**
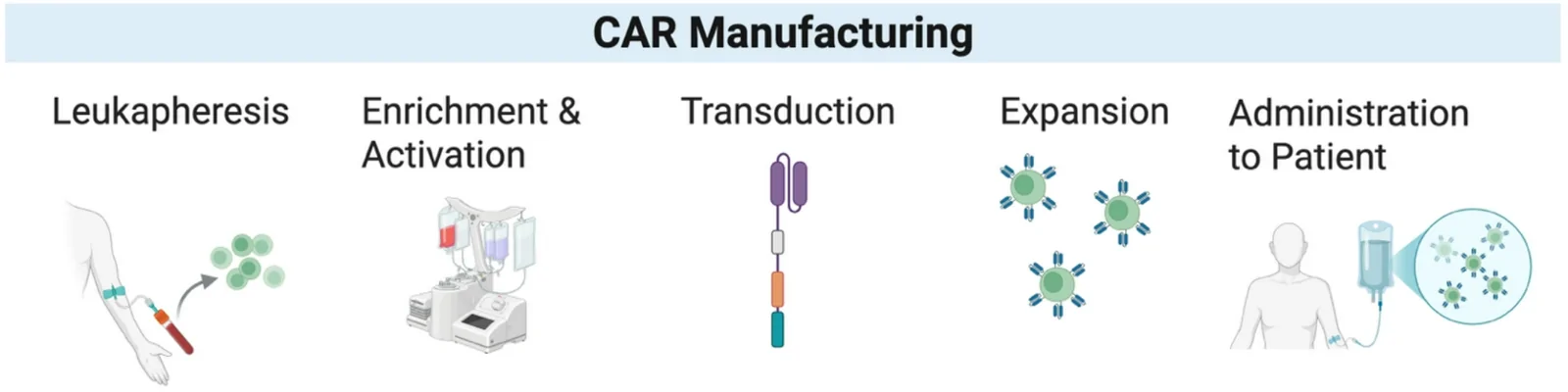
Simple CAR-T cell manufacturing pipeline. In the first step of leukapheresis, endogenous T cells are collected from the patient, often via drawing blood. Enrichment and activation involve selecting and stimulating the T cells of interest, which prepares them for transduction, or genetic modification. The genetically modified T cells are then expanded (i.e., replicated to increase numbers) and then administered to the patient. Created in BioRender. Anderson, J. (2026) https://BioRender.com/fpp7i3u

To develop CAR-T cells, endogenous T cells are taken from a patient and modified to recognize an antigen of interest. After expansion, they are infused back into the patient to identify the antigenic target and initiate an immune response [8] (Fig. 2). A breakthrough for treating haematological malignancies, it has proven difficult to extend encouraging CAR-T cell results to solid tumours. Among the challenges include immunosuppressive tumour microenvironments, various toxicities, CAR-T cell exhaustion, and tumour antigen heterogeneity. Many variables in CAR design and method of manufacturing impact on the functioning of the final product. Of particular relevance are the number and nature of costimulation domains, the respective number of CD4 and CD8 cells in the product, and the length and cytokine milieu of manufacturing expansion [9].

However, even with these challenges, promising findings have emerged in relation to adult CNS cancers [10–13], as well as paediatric solid tumours, particularly high-risk neuroblastoma [14]. The first proof of concept paediatric CNS tumour CAR-T trials targeted the antigen HER2, which also provided proof of concept for intravenous and intracerebroventricular delivery, respectively [15, 16]. Building on this, particularly encouraging are early clinical trial results that have been reported, including those from Stanford University NCT04196413, using disialoganglioside GD2-targeting CAR-T cells in the treatment of diffuse midline gliomas (DMG) in a paediatric population, in which 9 out of 11 patients had DIPG whilst 2 patients had spinal tumours. Median overall survival rate for patients reached 20.6 months–compared to less than a year by current treatment approaches in DIPG patients–with several patients experiencing clinical benefits and/or reductions in tumour volume. One patient remarkably demonstrated a sustained complete response [17]. Complementing these findings, Seattle Children’s Hospital NCT04185038 implemented a B7 homolog 3 (B7-H3)-targeting CAR-T treatment for DMG, seeing a median survival from diagnosis of 19.8 months for their treated patients, with three patients living through the conclusion of the study [18].

While both studies have provided field-advancing results, they differ in a range of areas, necessitating the question of what can be learned from these clinical experiences and other early findings. Among important variables to consider are the CAR-T cell route of delivery, presence of lymphodepletion, identified target antigen, and CAR engineering features. As of writing, there are 15 relevant clinical trials using CAR-T cells in the treatment of paediatric CNS cancers, all of which exhibit nuances in these variables (see Table 1).

**Table 1:** Active clinical trials

Study Name	Responsible Party	NCT Number	Disease	Target	Mode of Delivery	Lymphodepletion	Engineering Features
GD2 CAR T Cells in Diffuse Intrinsic Pontine Gliomas (DMG) & Spinal Diffuse Midline Glioma (DMG)	Stanford University	*04196413*	H3K27M-mutant Diffuse Intrinsic Pontine Glioma or H3K27M-mutant Spinal Diffuse Midline Glioma	GD2	IV followed by ICV	Yes	14g2a-CD8-BBz-iCasp9
C7R-GD2.CAR T Cells for Patients with GD2-expressing Brain Tumors (GAIL-B)	Baylor College of Medicine	*04099797*	**Cohort 1**: Diffuse Midline Glioma, High Grade Glioma, Diffuse Intrinsic Pontine Glioma, Medulloblastoma & other rare GD2-expressing brain cancers **Cohort 2**: Progressive Pontine Diffuse Midline Glioma, High Grade Glioma & Diffuse Intrinsic Pontine Glioma expressing GD2	GD2	IV only *or* IV followed by ICV	Yes	C7R gene
GD2-CAR T Cells for Pediatric Brain Tumours	Bambino Gesù Hospital and Research Institute	*05298995*	**Arm A**: Medulloblastoma & other embryonal tumours **Arm B**: Hemispheric High Grade Glioma **Arm C**: Thalamic High Grade Glioma, Diffuse Midline Glioma, Diffuse Intrinsic Pontine Glioma & other rare CNS tumors	GD2	IV	Yes	Third generation (4.1BB-CD28) with iC9
CAR T Cells to Target GD2 for DMG (CARMIGO)	University College London	*05544526*	Diffuse Midline Glioma	GD2	IV followed by ICV	Yes	Second generation (28-z)
Study of B7-H3-Specific CAR T Cell Locoregional Immunotherapy for Diffuse Intrinsic Pontine Glioma/​Diffuse Midline Glioma and Recurrent or Refractory Pediatric Central Nervous System Tumors	Seattle Children’s Hospital	*04185038*	**Arm A**: Non-Diffuse Intrinsic Pontine Glioma supratentorial tumors **Arm B**: Non-Diffuse Intrinsic Pontine Glioma infratentorial tumors or leptomeningeal tumors **Arm C**: Diffuse Intrinsic Pontine Glioma	B7-H3	IC *or* ICV	No	Second-generation, SCRI-CARB7H3
Loc3CAR: Locoregional Delivery of B7-H3-CAR T Cells for Pediatric Patients with Primary CNS Tumors	St. Jude Children’s Research Hospital	*05835687*	**Cohort 1**: Non-brainstem primary CNS tumours **Cohort 2**: Diffuse Midline Gliomas	B7-H3	ICV	No	CD28z signaling domain, 41BB ligand
Safety and Efficacy of Loco-regional B7H3 IL-7Ra CAR T Cell in DMG (CMD03DIPG)	Chulalongkorn University	*06221553*	Diffuse Intrinsic Pontine Glioma	B7-H3	ICV	Not mentioned	IL-7Ra signaling
CAR T Cells After Lymphodepletion for the Treatment of IL13Rα2 Positive Recurrent or Refractory Brain Tumors in Children	City of Hope Medical Center	*04510051*	CNS tumors (broadly)	IL13Rα2	ICV	Yes	Hinge-optimized, 41BB-co-stimulatory, CD19+ (IL13[EQ]BBzeta/CD19t+)
Genetically Modified T-cells in Treating Patients with Recurrent or Refractory Malignant Glioma	City of Hope Medical Center	*02208362*	Malignant Glioma	IL13Rα2	IT, IC, ICV *or* dual delivery of IT/IC and ICV	No	Hinge-optimized, 41BB-costimulatory, CD19+ (IL13Ra2-CAR/CD19t+)
HER2-specific CAR T Cell Locoregional Immunotherapy for HER2-positive Recurrent/​Refractory Pediatric CNS Tumors	Seattle Children’s Hospital	*03500991*	CNS tumours (exclusion of DMG)	HER2	IC *or* ICV	No	Second-generation
HER2-specific Chimeric Antigen Receptor (CAR) T Cells for Children With Ependymoma	Pediatric Brain Tumor Consortium	*04903080*	Ependymoma	HER2	IV	Yes	Second generation (28-z)
T Cells Expressing HER2-specific Chimeric Antigen Receptors (CAR) for Patients with HER2-Positive CNS Tumors (iCAR)	Baylor College of Medicine	*02442297*	Primary CNS tumours (exclusion of DMG)	HER2	IT, IC *or* ICV	Yes, depending on cohort	Second generation (41BB-z)
CMV-specific Cytotoxic T Lymphocytes Expressing CAR Targeting HER2 in Patients with GBM (HERT-GBM)	Baylor College of Medicine	*01109095*	Glioblastoma	HER2	IV	No	HER2-CD28 CMV-T
Study of B7-H3, EGFR806, HER2, And IL13-Zetakine (Quad) CAR T Cell Locoregional Immunotherapy for Pediatric Diffuse Intrinsic Pontine Glioma, Diffuse Midline Glioma, And Recurrent or Refractory Central Nervous System Tumors	Seattle Children’s Hospital	*05768880*	**Arm A**: Diffuse Intrinsic Pontine Glioma **Arm B**: Non-pontine Diffuse Midline Glioma & other CNS tumours	Multiple (B7-H3, EGFR806, HER2 & IL13-zetakine)	ICV	No	Second-generation
A Phase I Study of the Safety of Autologous GD2-Specific Chimeric Antigen Receptor-Expressing T Cells in Children with Diffuse Midline Glioma	Sydney Children’s Hospital	ACTRN12622000675729	DMG	GD2	IV initial with IV or ICV subsequent infusions	Yes	Third generation GD2-28-OX40-Zeta

ICV: intracereborventricular, IT intrattumoural, IC intracavity

Taken as a whole, these early clinical findings, combined with results of CAR-T cells for other cancers, represent an exciting opportunity warranting further exploration in treating some of the highest risk cancers in the paediatric population.

### The unique difficulty and opportunity of the brain

Within the solid tumour landscape, the brain poses a unique set of difficulties and opportunities for immunotherapy, largely because of the blood-brain barrier (BBB) and the CNS’s immunologically distinct nature. Adding a paediatric and developmental lens contributes further complexity.

The BBB plays an essential role in the maintenance of homeostasis: it serves as a tightly regulated interface for nutrients to cross into the CNS and simultaneous protector against pathogens [19]. The brain has historically been viewed as immunologically silent, stemming from early experiments documenting limited rejection of foreign tissue transplants [20, 21]. This finding raises an interesting question about the use of allogeneic T cells in CNS cancers, an approach which carries the advantage of being more readily available and cost effective [22]. The brain has recently become conceptualized as immunologically “distinct” [23]. Evidence for this classification includes documented traversing of immune cells into the brain. For example, leukocytes have been found to spread into the CNS via the choroid plexus, the source of cerebrospinal fluid (CSF) [24] and activated T cells are among those shown to traverse in this manner [25–27]. Initiated immune responses in the brain are tightly regulated because excessive inflammation and intracranial pressure risks life-threatening consequences. Indeed, there are remarkable homeostatic mechanisms [28], which are evolutionarily adaptive mitigators of risk, but also serve as an obstacle to immunotherapy. As such, CNS tumour sites display particularly immunosuppressive microenvironments such that traversing the BBB does not guarantee effective penetration of the tumour.

Significant tumour penetration comes with a different set of challenges in the form of toxicities. One of the primary risks of CAR-T cell treatment is cytokine release syndrome (CRS). Upon binding the target antigen, the initiated immune response involves recruitment of inflammatory cytokines. In CRS, many cytokines are circulated, initiating potentially aggressive systemic inflammation. Mild symptoms often include fever, and more severe symptoms can include hypoxia and hypotension [29]. CRS can precede immune-effector cell-associated neurotoxicity syndrome (ICANS). Inflammatory cytokines in the brain can interfere with normal functioning of the BBB with resulting generalized inflammation triggering such symptoms as headache and confusion through to seizure and coma. The prevalence of ICANS appears to be related to the dose of CAR-T cells and is likely to be affected by route of delivery with early reports that locoregional delivery to the brain can have the advantage of limiting CRS and ICANS [17]. The symptoms of CRS and ICANS are typically transient and reversible [30]. A third potential toxicity is tumour-inflammation associated neurotoxicity (TIAN). Considered on-tumour and on-target, TIAN involves localized inflammation at the tumour site [31]. Symptoms vary from headaches and fevers to severe hydrocephalus [32]. Prevalence of TIAN has been documented to trend downward as additional infusions are administered, with symptoms appearing reversible [17]. Recently, preclinical studies of CAR-T immunotherapy in both brain and extracranial tumours can lead to measurable cognitive deficits in experimental mice, reinforcing the importance of long-term follow-up in clinical studies [33]. Finally, a haemophagocytic lymphohistiocytosis (HLH) like syndrome has been increasingly recognized in patients receiving CAR-T cells for solid cancers, including those that do not have severe CRS. The term immune effector cell-associated HLH-like syndrome (IEC-HS) has been coined to describe this phenomenon [34].

The problem of neurotoxicities of CAR-T cells targeting brain tumours is compounded in paediatric oncology by the early developmental nature of the brain. Whilst neuroplasticity is especially relevant for the paediatric brain and might mitigate late effects, the role of inflammation in many neurological diseases [35] means trials to evaluate CAR-T in paediatric brain tumours must be carefully evaluated both for short-term and long-term neurological effects.

Taken together, CNS immunotherapy approaches face a complicated crux of simultaneously overcoming immunosuppressive mechanisms *and* mitigating risk resulting from an immune response. Striking this balance involves optimization of all the following variables.

### Routes of delivery

For haematological malignancies (i.e., leukemia and lymphoma), of which CAR-T has become a widely adopted treatment, CAR-T cells are intravenously injected and travel throughout the vascular system until target antigen recognition. Upon recognition, a proliferative CAR-T expansion is initiated [36].

**Fig. 3:**
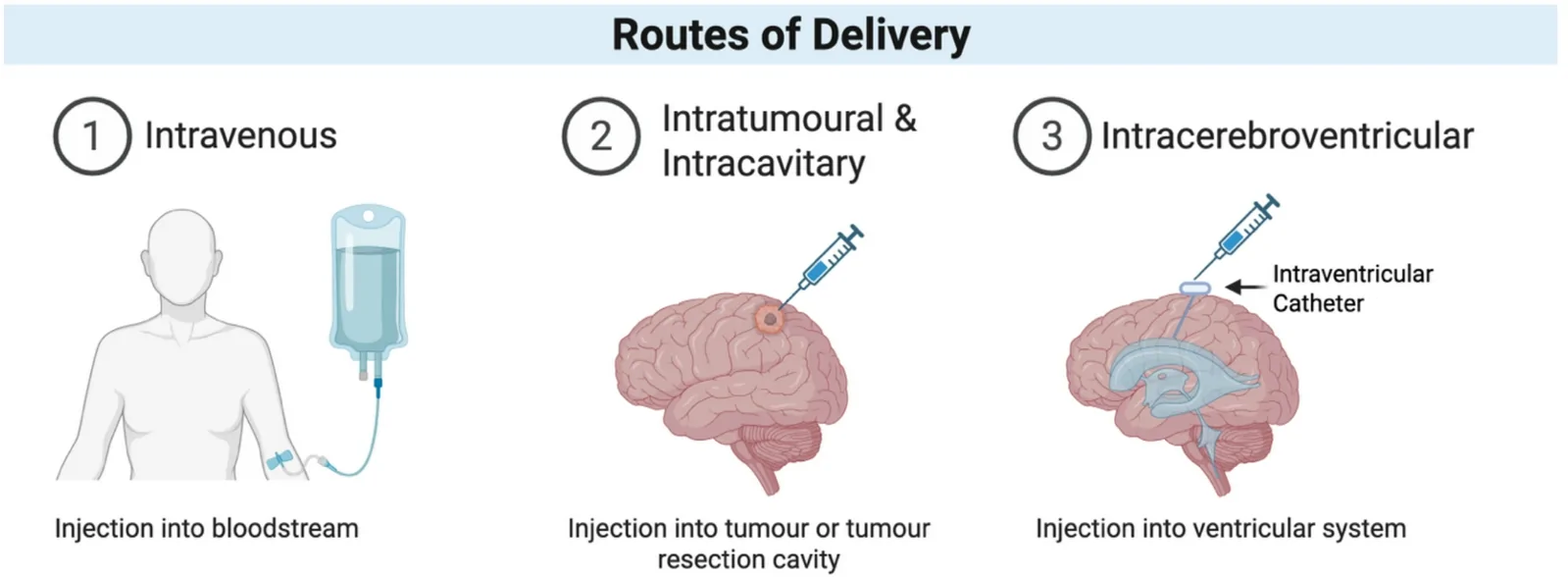
Three main routes of delivery for CAR-T cells into the CNS. Created in BioRender. Anderson, J. (2026) https://BioRender.com/b6w9iuz

Among the active clinical trials for CNS tumours, there are three predominant methods of delivery for CAR-T cells, with numerous trials employing a combination: intravenous, intratumoural/intracavitary, and intracerebroventricular (Fig. 3). Assessing the feasibility and safety of the manner of delivery is a necessary prerequisite to evaluating efficacy because risks and types of toxicity differ between these routes of administration.

Intratumoural (IT) and intracavitary (IC) delivery confers the benefit of direct delivery to the target site, mitigating risk of CAR-T cell exhaustion. It has already been safely used in clinical trials for breast cancer [37] and head and neck cancer [38], with early findings in brain tumours similarly proving encouraging in terms of tolerance [10, 39]. Intracerebroventricular (ICV) administration has also increasingly gained traction. ICV distributes CAR-T cells by exploiting the physiological flow of CSF through the CNS. Mouse models for CNS malignancies have built a strong evidence base for the efficacy of this approach [13, 40, 41], and several clinical trials in a paediatric population have shown this manner of delivery to be well-tolerated [17, 18]. Preclinical models have found ICV to confer benefits over IC, which both exhibited higher anti-tumour efficacy compared to IV administration [13, 41, 42].

In Stanford NCT04196413, all patients began with IV infusion. Early findings from the study found that even patients responding well to this initial injection risk progression 2–3 months later. Therefore, eligible patients demonstrating a positive response and/or stability proceeded to ICV infusions. At Seattle Children’s Hospital NCT04185038, patients exclusively received repeated ICV doses, which were deemed tolerable. Phase I results from these two trials point to the seeming safety of both the IV and ICV approach, but further data is needed.

### Lymphodepletion and patient preparation

Conditioning is an intervention to prepare a recipient for adoptively transferred T cells. Lymphodepletion is a widely used form of conditioning often achieved through using chemotherapy to deplete endogenous lymphocytes, creating space and homeostatic cytokines for transferred cells, inducing inflammation, and thereby aiding the engraftment of the CAR-T cells. The degree to which this “lymphodepletion” also functions by depletion of tumour-associated myeloid cells, including suppressive macrophages, MDSC and microglia in brain tumours, and the optimal timing of preparative chemotherapy regimens are important areas of research interest. Lymphodepletion is now universally recognized as a requirement for systemic delivery in adoptive T cell transfer [43, 44].

However, as lymphodepletion provides suppression of lymphocytes, patients become susceptible to serious viral infections. In contrast, the approach of repeated infusions in the absence of lymphodepletion mitigates the infection risk and might be a preferred approach for ICV delivery. Among the 15 total clinical trials, eight have explicitly included lymphodepletion conditioning. As the outcomes of these trials are reported, key insights into its requirement are likely to emerge. Moreover, there are numerous approaches to delivering lymphodepletion (i.e., chemotherapy, radiation, antibodies) [45] and further studies are required to identify the most optimal strategies. Stanford NCT04196413 used fludarabine and cyclophosphamide prior to delivery of the initial IV infusion (i.e., systemic delivery) but did not lymphodeplete prior to the initial ICV injection, and Seattle Children’s Hospital NCT04185038 did not use lymphodepletion for their repeat ICV infusions.

A potentially critical variable in relation to patient response is tumour disease burden at time of CAR infusion. As well as the greater risk of toxicities related to local inflammation with large tumours in eloquent areas of the brain, there is both clinical and preclinical evidence of greater disease control associated with smaller disease burden [10, 15, 46, 47]. Hence there is strong rationale for timing of preparative surgical debulking to be sufficiently close to CAR-T infusion to maximise response whilst allowing sufficient time for resolution of complications associated with surgery.

### Antigenic targets

One of the challenges in the application of immunotherapy to brain tumours is identifying an appropriate target antigen. An ideal target is consistently expressed in tumour cells but exhibits minimal expression in healthy cells so that the activated immune cascade remains as localized to the cancer as possible. For the treatment of paediatric CNS cancers, five antigens are the main targets under investigation:

### Disialoganglioside GD2 (GD2)

A well-studied tumour-associated antigen, GD2 is a glycosphingolipid that plays a wide range of roles in oncogenesis including cell adhesion, migration, proliferation, and apoptosis resistance [48]. This surface antigen is expressed on healthy tissue in peripheral nerves, as well as multiple areas in the CNS, but in low enough levels to still make it a suitable target [49]. GD2 is currently being explored for other solid tumours, particularly neuroblastoma where there have already been encouraging findings treating with monoclonal antibodies leading to the approval of dinutuximab [50, 51], as well as with CAR-T cells [14, 52]. These results prompt the hopeful application to GD2-positive tumours in the CNS.

As discussed above, the Stanford clinical trial NCT04196413 CAR targeted GD2 [53, 54]. Their findings will provide the foundations for other trials investigating analogous approaches: the GAIL-B trial NCT04099797 similarly administers CARs via IV followed by ICV but has added a gene to prolong T cell survival. Bambino Gesú Hospital in Rome (NCT05298995) administers via IV and has added a suicide gene for deployment in the presence of severe toxicity [55]. CARMIGO NCT05544526 at Great Ormond Street Hospital in London is using a CAR design previously reported on in neuroblastoma [56].

### B7 Homolog 3 (B7-H3)

Another widely studied antigenic target, B7-H3 (CD276) is an immune checkpoint molecule documented to play a critical role in tumour progression through fostering an immunosuppressive tumour microenvironment. With limited expression in healthy tissue, B7-H3 is present in high and consistent levels across a range of cancers [57], including many paediatric brain tumours [58, 59]. This expression pattern makes it an enticing antigenic target, with monoclonal antibody [60] and CAR-T cell therapies [61] currently being explored in numerous tumour types. Encouraging preclinical results have been reported for glioma [58], medulloblastoma [59], and glioblastoma [62].

As discussed above, phase I findings from the Seattle Children’s Hospital clinical trial NCT04185038 using a B7-H3 CAR found a significant improval in survival rates [18]. Several additional ongoing clinical trials involve this antigenic target (NCT04185038, NCT05835687, NCT06221553, NCT05768880).

### Interleukin 13 receptor subunit alpha 2 (IL13Rα2)

A cytokine with a well-documented role in allergic reactions and anti-inflammatory responses, IL-13 is an important immunoregulatory molecule. Among other receptors, IL-13 binds to IL13Rα2, which is minimally expressed in healthy tissue, upregulated in certain diseases (e.g., parasitic diseases, inflammatory bowel disease), and notably highly expressed in various cancers [63]. While not as uniformly expressed across paediatric brain cancers in comparison to other antigenic targets (i.e., GD2, B7-H3), IL13Rα2 has a well-documented association with poor prognosis across a wide range of cancers. Elevated expression of this receptor has been identified in breast cancer [64], lung cancer [65], ovarian cancer [66], pancreatic cancer [67], melanoma [68], colorectal cancer [69], and glioblastoma [70].

City of Hope Medical Center leads the development of an IL13Rα2 CAR (NCT04510051, NCT02208362) with an IL-13 zetakine CAR that preferentially binds to IL13Rα2 over IL13Rα1. There has been encouraging anti-tumour activity in preclinical studies [42, 71]. Phase I trial results in high grade glioma demonstrated the safety of their approach, with 50% of patients (primarily adults) exhibiting stable or improved disease, and one patient showing a complete response [10].

### Human epidermal growth factor receptor 2 (HER2)

HER2 belongs to a family of receptors that facilitate cellular growth and differentiation by binding tyrosine kinase. In cancerous malignancies, HER2 promotes tumour expansion [72]. Similar to other antigenic targets, it exhibits relatively low expression in healthy tissues but shows over-expression in cancers. HER2-targeting immunotherapy has demonstrated field-changing efficacy in treating a particular type of breast cancer [73]. In CNS contexts, preclinical results of a human xenograft model of HER2 CAR-T cell treatment of breast cancer metastases in the brain showed a robust anti-tumour effect [40].

In a ranked expression hierarchy of five antigens in paediatric brain tumours (B7-H3, GD2, IL13Rα2, EphA2, HER2), HER2 co-ranked with EphA2 as the least optimal [58]. However, findings focussed on specific sub-types of paediatric CNS cancers have documented a more consistent over-expression, including in ependymoma [13] and glioblastoma [74], with lower HER2 levels related to longer survival time. Preclinical findings for DMG showed HER2 CARs to significantly reduce tumour burden [75]. Several active clinical trials are further investigating HER2 (i.e., NCT03500991, NCT04903080, NCT02442297, NCT01109095), which will provide further information on the validity of HER2 as a target in paediatric CNS tumours.

### Epidermal growth factor receptor (EGFR) variations

Similar to HER2, EGFR is a tyrosine kinase receptor that plays an important role in cellular proliferation [76]. There is a documented upregulation of EGFR [77], as well as mutation in the form of EGFRvIII (deletion of exons 2–7), in glioblastoma [78, 79]. The specificity of this mutation to tumour cells makes EGFRvIII an attractive target. However, extension of these relatively consistent expression levels to other tumour types has been inconclusive [80], and the relationship between EGFRvIII positivity and survival rate is also unresolved [81]. A phase I trial of anti-EGFRvIII CAR-T cells in adults found no efficacy [82], and as of writing, while there is ongoing investigation of EGFRvIII in adults with glioblastoma (ISRCTN22366199), there are no active clinical trials in the paediatric population that involve EGFRvIII as the identified antigen of interest. A CAR targeting a different epitope of EGFR that is not accessible on healthy tissue but prominent on tumour cells overexpressing EGFR has been developed [83]. This CAR has been assessed in a phase I trial of EGFR-positive paediatric CNS tumours (NCT03638167), but limited results have been published.

### Combining antigenic targets

Antigen escape has been documented in multiple haematological studies [84, 85], combined with heterogeneity of antigen expression has fuelled the proposed solution of targeting multiple antigens [86]. A phase I trial using bivalent CAR-T cells in the treatment of adult glioblastoma has reported preliminary safety of this approach [87], but it requires validation. Seattle Children’s Hospital NCT05768880 is currently exploring the use of a “quad” CAR-T cell that targets B7-H3, IL13, HER2, and EGFR806 for treatment of DMG and other CNS tumours.

### Preclinical advancements in cell therapy for paediatric brain tumours

#### Target selection

**Fig. 4:**
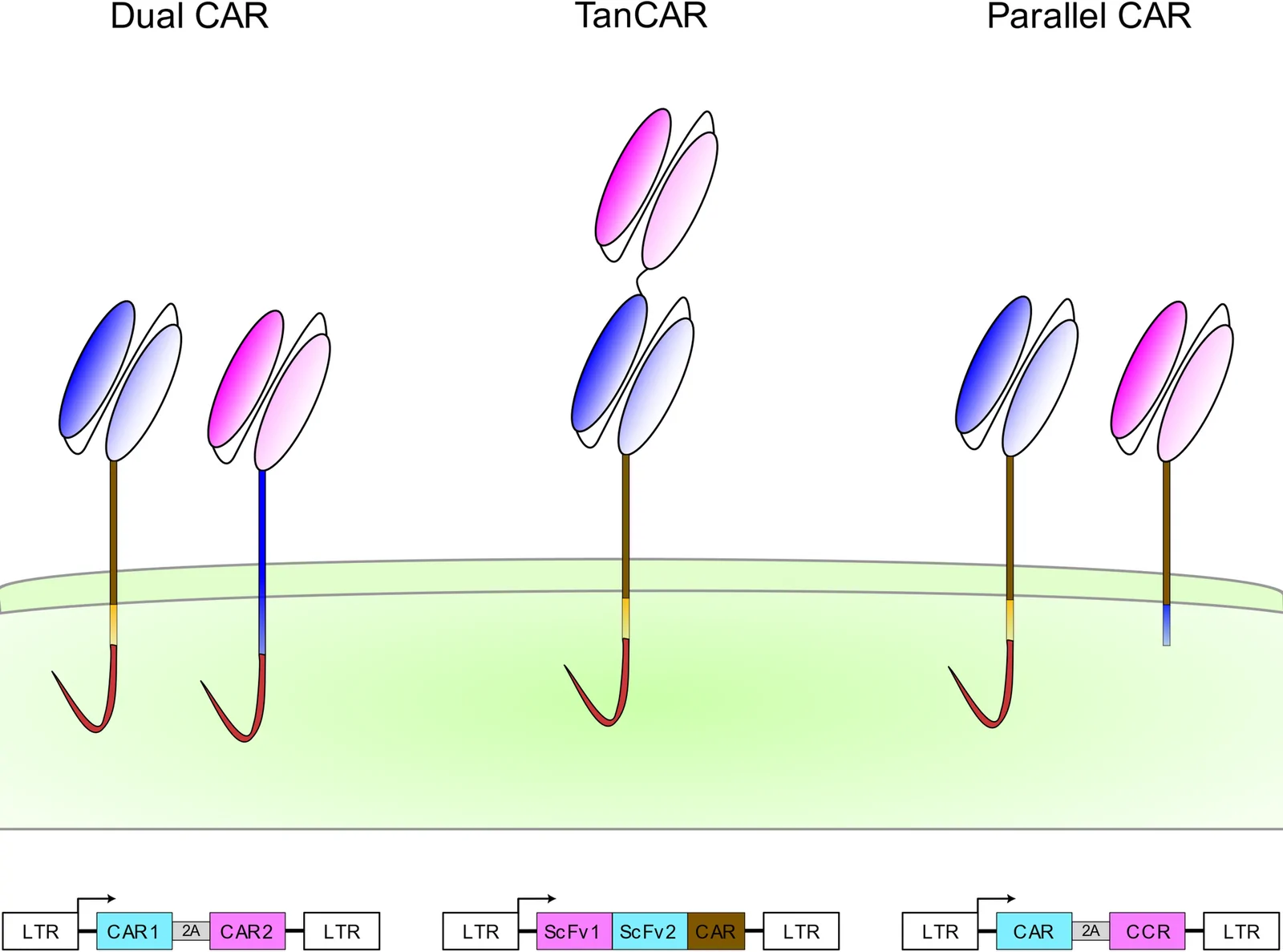
Multi-antigen targeting CAR T-cells. Top shows a cartoon with a schematic of the transgene shown underneath. Dual CAR consists of two second-generation CARs targeting different antigens independently. TanCAR has two scFvs expressed together. Parallel CAR has one full-length CAR co-expressed with a Chimeric Costimulatory Receptor (CCR) that provides additional costimulatory signaling

The trials described above show the narrow range of antigens that are currently being developed for CAR T-cell therapy of paediatric brain tumours. To elaborate on the multi-antigenic approach, many clinical and preclinical studies in haematological malignancies use CARs designed to target multiple antigens in an “OR” gate fashion (i.e., CAR-T cells activate when one of the targets is present) using various methods of achieving multi-CAR expression, including multi-transduction [88–90], multi-cistronic constructs [87, 91–93]or loop/tan CAR designs [94] (Fig. 4). Multi-transduction requires two or more clinical-grade vectors to be produced and results in heterogenous populations of T cells that contain variable proportions of each CAR. Multi-cistronic vectors solve this by only using one vector, reducing manufacturing costs. However, increasing the size of the transgene, especially in the current standard of viral-mediated delivery, can reduce levels of transduction and realistically only allows for dual antigen targeting constructs [95]. This also reduces room for extra modules that can be incorporated for either safety, such as iCas9 suicide gene [96, 97], or “armouring” to improve efficacy. Use of parallel CAR designs [98–101] can reduce the size of the construct, with only one CAR requiring CD3ζ. Tandem or Loop CAR designs can reduce the size of the transgene even further; however, scFv stability is a concern when multiple are expressed in the same protein [102]. In addition the epitope that each binder recognises on the target antigen needs to be taken into consideration because binder for a distal epitope may result in an inefficiently formed immune synapse [103, 104], increasing the difficulty in optimising these designs. The above designs all function with OR-gate logic which can prevent antigen escape, however finding one, let al.one multiple tumour-specific antigens can be challenging. Therefore, there is movement towards using tumour-associated antigens and engineering CARs to act with AND or NOT gate logic to reduce the risk of on-target off-tumour activity. Introducing more refined logic into CAR T-cell engineering can allow a higher degree of specificity, making CARs responsive to three or more inputs with various combinations of logic [105]. Though this level of engineering has yet to be applied to CAR T-cells treating paediatric CNS tumours, there is a desire for treatments to have more sophistication, especially in areas as sensitive as the brain where unwanted damage can have serious long-term developmental issues. An alternate strategy for minimising on-target off-tumour toxicity risk is engineering the CAR-T cells for only transient expression of the CAR, for example through electroporation with mRNA encoding the receptor [106].

#### CAR armours

**Fig. 5:**
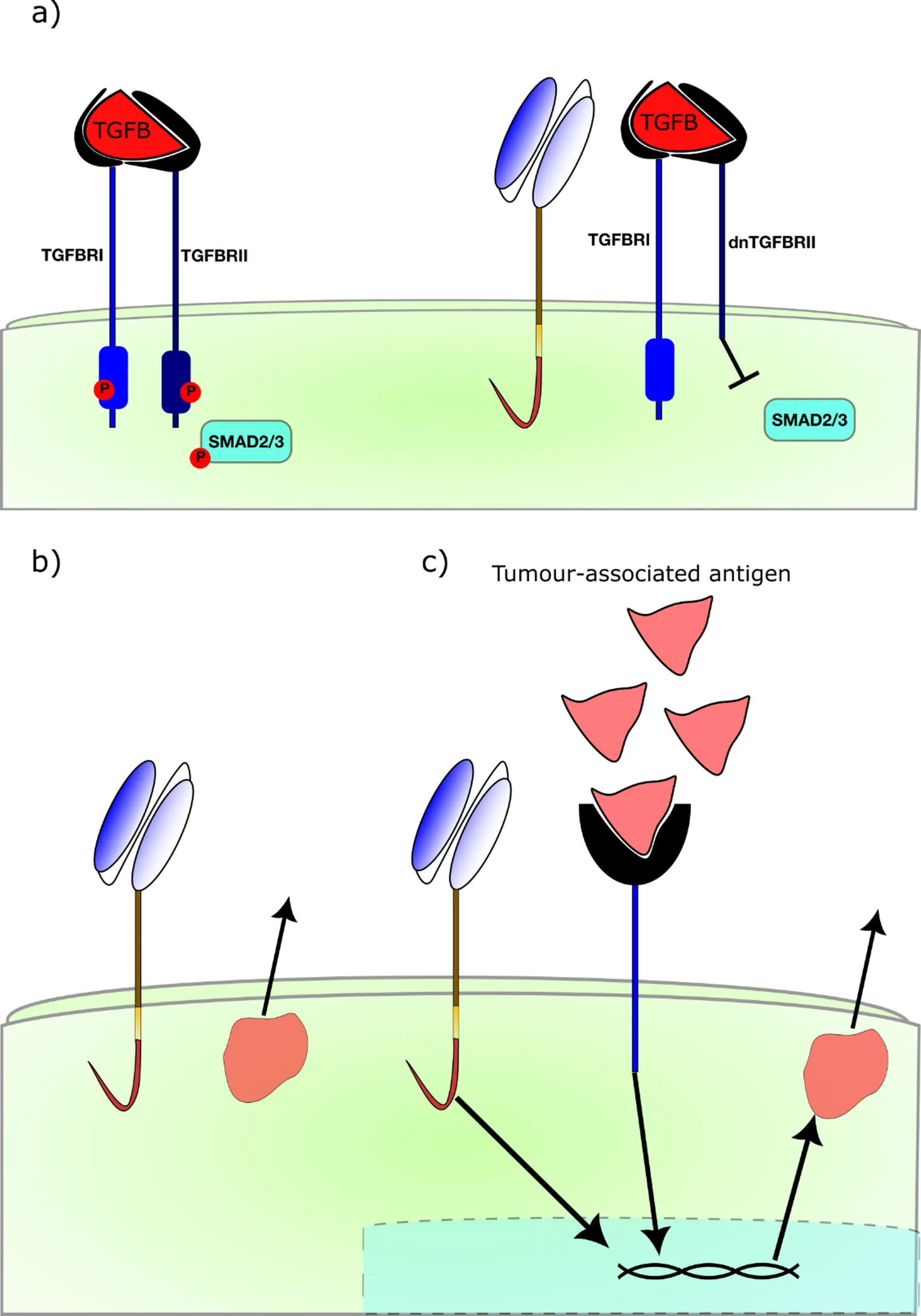
Examples of CAR Armours. Armoured CARs consist of a standard second-generation co-expressed with a complementary module. **(A)** TGFβ signals through TGFBRI and TGFBRII, co-expressing a dominant negative TGFBR prevents immunosuppressive effects of TGFβ on the CAR T-cell. **(B)** CARs can co-express a cytokine or other secreted protein to effect tumour or the TME. **(C)** Constitutive cytokine production can be toxic, coupling this with some logic-gated signaling allows expression only in the presence of a specific antigen (e.g., tumour-associated) or on CAR activation

The main short-coming of CAR T-cells applicability to solid tumours, including paediatric brain tumours, is their lack of persistence and inability to survive and proliferate in the immunosuppressive tumour microenvironment [107]. As well as drug treatments to target suppressive cells in the tumour environment (TME), armouring CARs with extra modules that promote T-cell persistence, survival and efficacy can bestow increased resistance to the immunosuppressive effects of the TME (Fig. 5). An EGFRvIII CAR co-expressing a dominant negative version of TGFβR (dnTGFβR) showed efficacy in preclinical models of glioma [108] and CAR T-cells with dnTGFβR have entered clinical assessment in adult solid tumours [109], making future translation to paediatric tumours likely. Another logical armour has CAR T-cells producing pro-inflammatory cytokines, however systemic cytokine expression can result in high levels of toxicity [110, 111], this has led to innovative designs that can control expression of these cytokines both spatially and temporally, or in response to certain tumour or TME antigens in order to reduce the likelihood of toxicity [112]. Recent clinical experience in extracranial tumours targeting GPC3 reports improved clinical responses compared with the historical non-armoured equivalent [113].

### CAR design

#### Safety switches

**Fig. 6:**
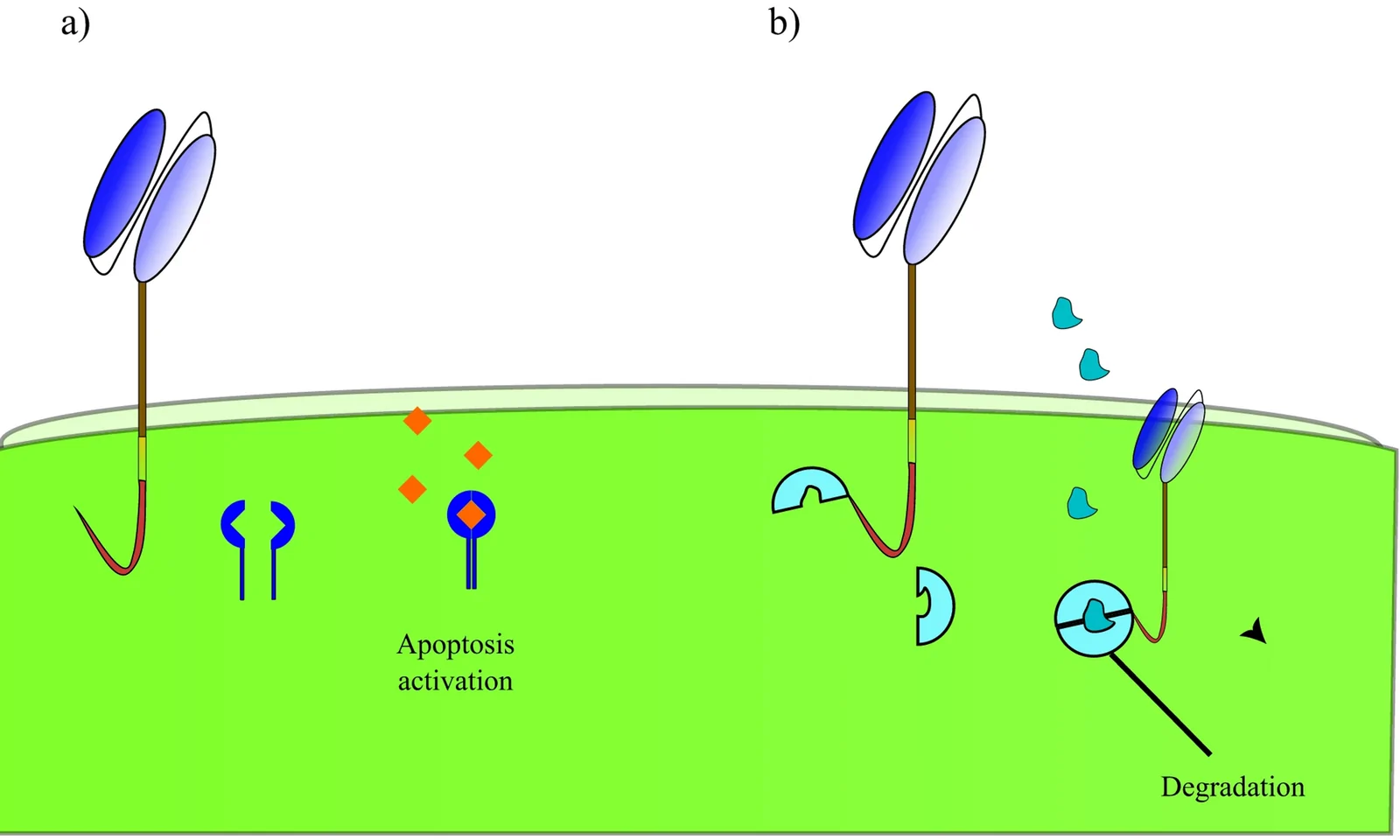
CAR safety switches. **(A)** Suicide genes activate apoptosis, e.g. iCas9, results in CAR-T cell death upon activation of the domain using exogenous small molecules. **(B)** Temporary CAR degradation can be achieved using degrons whereby a degradation domain is expressed on the CAR and administration of drugs such as IMiDs results in CAR degradation resulting in temporary abrogation of CAR activity

Inflammation in the brain can cause serious adverse events such as oedemas and encephalopathy and has been shown to be a side-effect of CAR-T treatment [114–116]. Therefore, the addition of suicide genes and context switches is a necessary consideration in CAR design as they can reduce or even prevent these adverse events (Fig. 6). Suicide genes allow CARs to be inhibited or killed by the addition of exogenous drugs at signs of excessive toxicity. However, current iterations that are in trial are irreversible; a more elegant method of addressing safety concerns is to engineer the CAR to only express in the presence of tumour antigen, reducing the likelihood of toxicity whilst maintaining the ability to turn the CAR back on [117–120]. Recent work has also looked to reversibly control CAR signaling through clinically available drugs such as dasatinib [121] or reduce expression through the use of exogenous drugs, for example the use of IMID-responsive domains has recently gained traction in CAR design [122], allowing reversible downregulation of the CAR whilst maintaining an anti-tumour effect.

#### Production

The lack of persistence of CAR T-cells in treating paediatric brain tumours is apparent, with preclinical brain tumour models showing short-term expansion followed by a drastic drop in numbers, with some studies implanting intracranial cannulas for repeat administration which increases both the technicality and invasiveness of the studies [123]. Current autologous CAR T-cells have a long lead time (2 weeks) from leukapheresis to cell manufacture, and for multiple infusions will likely require multiple blood draws and multiple manufacturing cycles which not only adds time drain but also adds costs [124]. One of the methods to circumvent both lead time and cost is the development of allogeneic CAR T-cells, i.e. CAR T-cells derived from donor blood which can be used in an off-the-shelf manner. Allo CAR-T are currently in clinical trials (NCT03692429) [125] and have shown efficacy in preclinical assessments [126]. Translation of these models to brain tumours would allow for multiple infusions without manufacturing lead time, however the efficacy in clinical trials has been limited due to host-vs-graft effect resulting in clearance of the CAR T-cells [127], therefore there is a need for further optimisation of allo-derived products.

#### Transduction

Recent studies have suggested a small risk of developing T-cell leukaemias post CAR T-cell infusion, which may be driven by the presence of viral sequences in the CAR T-cell expression cassette [128]. Though these findings are contentious [129, 130], there is still a desire to reduce the presence of foreign sequences in the CAR transgene which has led to a push to develop non-viral transduction methods [131]. While not brain tumour specific, this could allow an increased packaging capacity, resulting in the inclusion of more modules into the CAR T-cell and allowing the addition of all the mentioned innovative engineering strategies.

#### Trafficking

Though locoregional administration of CAR T-cells to brain tumours is relatively straightforward, the occasional presence of extra-CNS metastases mean that it is beneficial for CAR T-cells to be able to cross the blood-brain barrier in both directions. Recent work has shown that CNS-specific expression of a payload from an engineered cell into the CNS is possible from systemically administered cells [119] and some reported clinical responses in DMG have followed systemic delivery [17]. Several recent trials for these reasons have combined both systemic and locoregional delivery. Trafficking to the tumour site following systemic delivery can also be enhanced through engineering, for example co-expression of chemokine receptors [132–134].

### Alternative CAR cell types

While CAR T-cells have shown drastic responses in haematological malignancies, T cells are generally resident in the circulatory system, therefore there should be a focus on using tissue-resident cells for targeting solid tumours and brain tumours. In addition to this, identification of immune cells that are present in the tumour microenvironment, or those that are conspicuous by their absence, may give an insight into the “best” cell to use to cause anti-tumour regression.

#### Macrophages/microglia

In the context of brain tumours, microglia are an obvious target as they are the brain’s tissue-resident macrophages. Recent studies have shown that using CAR Macrophages (CAR-Mac) has resulted in promising preclinical results including in brain tumour models. In addition, initial solid tumour phase I results show high levels of safety [135]. One advancement that could be combined with this is that of in situ transduction. Indeed, recent work has shown that LNPs containing mRNA can transduce macrophages in situ [136] and, as microglia are the most abundant immune cell in the brain, this could lead to in situ transduction of microglia to repolarise or even target tumour cells.

#### Natural killer cells

NK cells have also emerged as a promising candidate for CAR engineering, with a number of trials showing efficacy in haematological malignancies [137], as well as non-engineered ex vivo expanded NK adoptive therapy being assessed in paediatric brain tumours showing safety and some response, albeit without significant persistence of adoptively transferred cells or clinical response [138]. This paves the way for CAR-NK cells in paediatric brain tumours, with preclinical models showing efficacy with GD2 [139] and B7-H3 [140] targeted CAR-NKs.

#### Preclinical modelling of CAR T-cells

One of the main drawbacks in current cell therapy research is the lack of translation from preclinical to clinical results. There are many preclinical animal studies that show outstanding anti-tumour efficacy with CAR T-cells against solid tumours that fail when they get to the clinic, with no solid tumour approved CAR T-cell therapies despite decades of positive results for preclinical results. One reason for this discrepancy is the use of immunodeficient mice which fail to capture the complex tumour microenvironment, especially when using models derived from cell lines. PDX models of paediatric brain tumours have shown some promise in recapitulating actual tumour biology [141–143], however there will still be a lack of tumour-immune interaction which is one of the main drivers of resistance to CAR T-cell therapy and as such is still a limited model. Approaches such as implantation of tumours in tissues representing the natural site of tumourigenesis (orthotopic implantation) has been regarded as favourable for inducing a more representative tumour environment. However, such models still typically fail to predict human clinical experience. A major factor for the mouse/human mismatch of result might be the much higher T cell dose per kg used in mice, which would be impractical to attain in parallel human studies.

One alternative to represent a more physiological tumour environment is to use genetically engineered mouse models (GEMMs) which have been shown to mirror human tumours. There are many validated GEMM models of paediatric brain malignancies, including medulloblastoma [144], high grade glioma [145], ependymoma [146] and diffuse midline glioma [147]. Whilst they have proven a valuable tool for understanding tumour biology, using these models in a translational research manner can be difficult in terms of technicality, logistics and translation. The main issues arise from the fact that most CARs use scFvs derived from mouse antibodies as their binding moieties, meaning that they are unlikely to be as reactive to the equivalent mouse target, if they even bind at all. This can be partially addressed through the production of transgenic mice, knocking in the human gene into the mouse locus [148], however this is highly dependent on the human protein being close enough to the mouse protein to not have an effect on normal tissue development. Another issue arises from effector cells needing to be obtained from mouse spleens and engineered with murinised versions of a CAR. Mouse splenocytes are much less amenable to ex vivo engineering and expansion compared to human PBMC-derived T-cells [149], however there have been advances made in generating and expanding mouse CAR T-cells [150, 151]. A further issue specific to brain tumour models is the stage at which the tumours are treated. In the current clinical state, CARs will likely be given after surgery and radiotherapy and whilst there are ways of administering directed radiotherapy to mice which have resulted in recurrent models of tumours [152, 153], surgical resection of tumours is an extreme technical challenge and the generation of metastatic models that recapitulate human metastatic disease are limited. Use of larger animal models might provide an opportunity for clinically relevant surgery and CAR-T combination studies but come with hurdles of expense and regulatory burden.

### The future of CAR T-cells in paediatric brain tumours

Paediatric brain tumours have offered a promising target for CAR therapy due to the ability to deliver the therapy locoregionally, mitigating any need for CAR T-cells to traffic to the tumour and cross the blood-brain barrier. Initial clinical results have been promising, however most responses have required multiple infusions of CAR T-cells, increasing the manufacturing costs and, for the most part, resulting in transient responses. This highlights a need for more innovation in improving CAR T-cell persistence and efficacy, however even with this efficacy there is a high likelihood that CAR T-cell therapy alone may not be enough to result in sustained long-term responses. Therefore, identifying optimal combinations of CAR T-cells and other targeted therapies may be the way forward for success in this devastating group of diseases.

## Data Availability

No datasets were generated or analysed during the current study.
